# Arteriovenous Fistula Simulating Deep Vein Thrombosis Following Lumbar Arthrodesis

**DOI:** 10.5334/jbsr.3731

**Published:** 2024-09-10

**Authors:** Gary Amseian, Tomás Fernández, Jaime Isern-Kebschull

**Affiliations:** 1 Department of Radiology, Hospital Clínic de Barcelona; 2 Department of Radiology, Hospital Clínic de Barcelona; 3 Department of Radiology, Hospital Clínic, University of Barcelona

**Keywords:** iatrogenic arteriovenous fistula, post-operative, deep vein thrombosis, arthrodesis, angio CT, emergency radiology

## Abstract

*Teaching point:* Although deep vein thrombosis is a common concern after lumbar arthrodesis, rare complications such as an iatrogenic arteriovenous fistula can present similarly, highlighting the importance of a broad, differential diagnosis and appropriate imaging for timely management.

## Case Report

**Figure 1:**
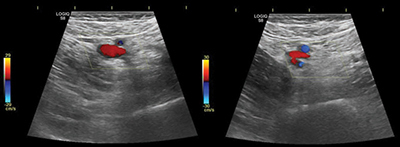
Doppler ultrasound of the left lower limb without signs of DVT.

A 70-year-old woman underwent an L4–L5 lumbar arthrodesis procedure without immediate complications. On the fourth post-operative day, however, she presented with painful swelling of the left lower limb. Given the recent surgery and immobilization, deep vein thrombosis (DVT) was suspected, and a Doppler ultrasound was performed. However, the ultrasound did not reveal any radiologic signs of DVT (Figure 1).

**Figure 2:**
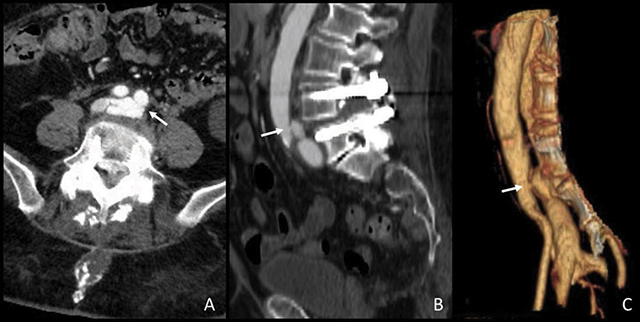
Angio-CT revealing an arteriovenous fistula involving the left common iliac vessels.

Due to the lack of evidence for DVT, a contrast-enhanced computed tomography (CT) scan was performed, which revealed an arteriovenous fistula (AVF) involving the left common iliac vessels (arrow in Figure 2).

**Figure 3:**
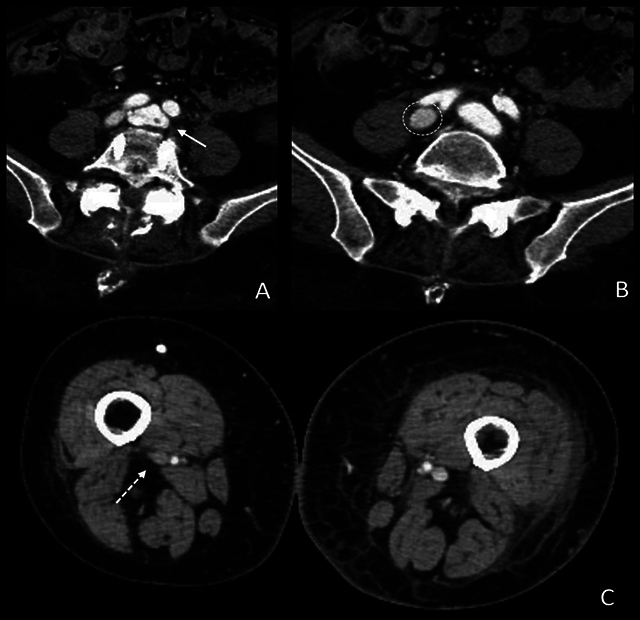
CT revealed arterial opacification of the left femoral vein and a swollen left lower limb.

The lumbar arthrodesis screws were visualized, and an irregularity of the left common iliac vein was noted, suggesting laceration (arrow in Figure 3, A). In the arterial phase, the iliac arteries and the left common iliac vein demonstrated intense enhancement, while the right common iliac vein showed normal mild enhancement (circle in Figure 3, B). The left lower limb was significantly swollen with subcutaneous edema, and contrast enhancement in both the femoral artery and vein was apparent, unlike in the contralateral leg, which showed no contrast in the right femoral vein (dashed arrow in Figure 3, C).

Subsequently, an aortic arteriography was performed, confirming the AVF with early filling of the left common iliac vein through the ipsilateral common iliac artery. Covered stents were successfully placed to treat the AVF, with good angiographic results. The patient recovered without complications and was discharged three days later.

## Comment

While deep vein thrombosis is a common concern in the post-operative setting, rare complications such as iatrogenic arteriovenous fistula can present with similar symptoms. Similar vascular complications have been reported in spinal procedures such as intervertebral disc surgery or lumbar reconstruction, but their incidence is very low, particularly with a posterior approach [1]. This case highlights an exceedingly rare complication following a posterior-approach lumbar arthrodesis, which, to our knowledge, has not been previously described. The clinical presentation of such an AVF can mimic that of DVT, underscoring the importance of considering a broad, differential diagnosis in post-operative patients.
